# Nonlinear associations between the ratio of family income to poverty and all-cause mortality among adults in NHANES study

**DOI:** 10.1038/s41598-024-63058-z

**Published:** 2024-05-26

**Authors:** Hong Yi, Minghui Li, Youzheng Dong, Zumao Gan, Lei He, Xiaozhong Li, Yu Tao, Zirong Xia, Zhen Xia, Yumei Xue, Zhenyu Zhai

**Affiliations:** 1 Department of Cardiology, Guangdong Cardiovascular Institute, Guangdong Provincial People’s Hospital, Guangdong Academy of Medical Sciences, Guangzhou, China; 2 Department of Cardiovascular Medicine, Fuwai Hospital, National Center for Cardiovascular Diseases, Chinese Academy of Medical Sciences and Peking Union Medical College, Beijing, 100037 China; 3 Department of Cardiovascular Medicine, The Second Affiliated Hospital of Nanchang University, Nanchang, 330006 China

**Keywords:** Medical research, Epidemiology

## Abstract

Socioeconomic status (SES) has been linked to mortality rates, with family income being a quantifiable marker of SES. However, the precise association between the family income-to-poverty ratio (PIR) and all-cause mortality in adults aged 40 and older remains unclear. A cross-sectional study was conducted using data from NHANES III, including 20,497 individuals. The PIR was used to assess financial status, and various demographic, lifestyle, and clinical factors were considered. Mortality data were collected from the NHANES III linked mortality file. The study revealed a non-linear association between PIR and all-cause mortality. The piecewise Cox proportional hazards regression model showed an inflection point at PIR 3.5. Below this threshold, the hazard ratio (HR) for all-cause mortality was 0.85 (95% CI 0.79–0.91), while above 3.5, the HR decreased to 0.66 (95% CI 0.57–0.76). Participants with lower income had a higher probability of all-cause mortality, with middle-income and high-income groups showing lower multivariate-adjusted HRs compared to the low-income group. This study provides evidence of a non-linear association between PIR and all-cause mortality in adults aged 40 and older, with an inflection point at PIR 3.5. These findings emphasize the importance of considering the non-linear relationship between family income and mortality when addressing socioeconomic health disparities.

## Introduction

Addressing socioeconomic health inequities has received a lot of attention in the field of public health^1,2^. Several epidemiological studies have demonstrated, across various populations, that socioeconomic status (SES) is significantly associated with mortality range^3–5^. Income, education, job, and neighbourhood features are all markers of SES and are correlated with mortality rates^6,7^. Additionally, these factors could affect mortality independently. Thus, analyzing the impact of each SES indicator on mortality is crucial to comprehending the precise purpose of socioeconomic status differences in mortality rates.

Some previous studies have investigated the relationship between each SES indicator (education, job) and mortality^8,9^. Nevertheless, disease studies find limited utility in employing educational and occupational stratifications as these models overly prioritize schooling and job placements while disregarding individuals' health-acquiring and purchasing capabilities^10,11^. Indexes of education and work are particularly challenging since they only specify aspects of these fields both within and across peers^10,11^. Family income is one of the most easily quantifiable of these factors, which can be adjusted according to the family income-to-poverty ratio (PIR)^12^. The PIR index of socioeconomic standing is potentially a more reliable indicator of socioeconomic status compared to education and occupation.

Insufficient data exists regarding the relationship between PIR (a validated measure of income inequality) and the overall impact on mortality rates. Nevertheless, research does suggest that individuals with a lower PIR have a higher vulnerability to all-cause mortality^13–15^. A cross-sectional study was conducted to establish an association between the PIR and the total mortality rate among individuals aged 40 and above. In order to ensure precision, we considered factors such as demographics, lifestyle, and clinical indications.

## Methods

### Study design and population

**Figure 1:**
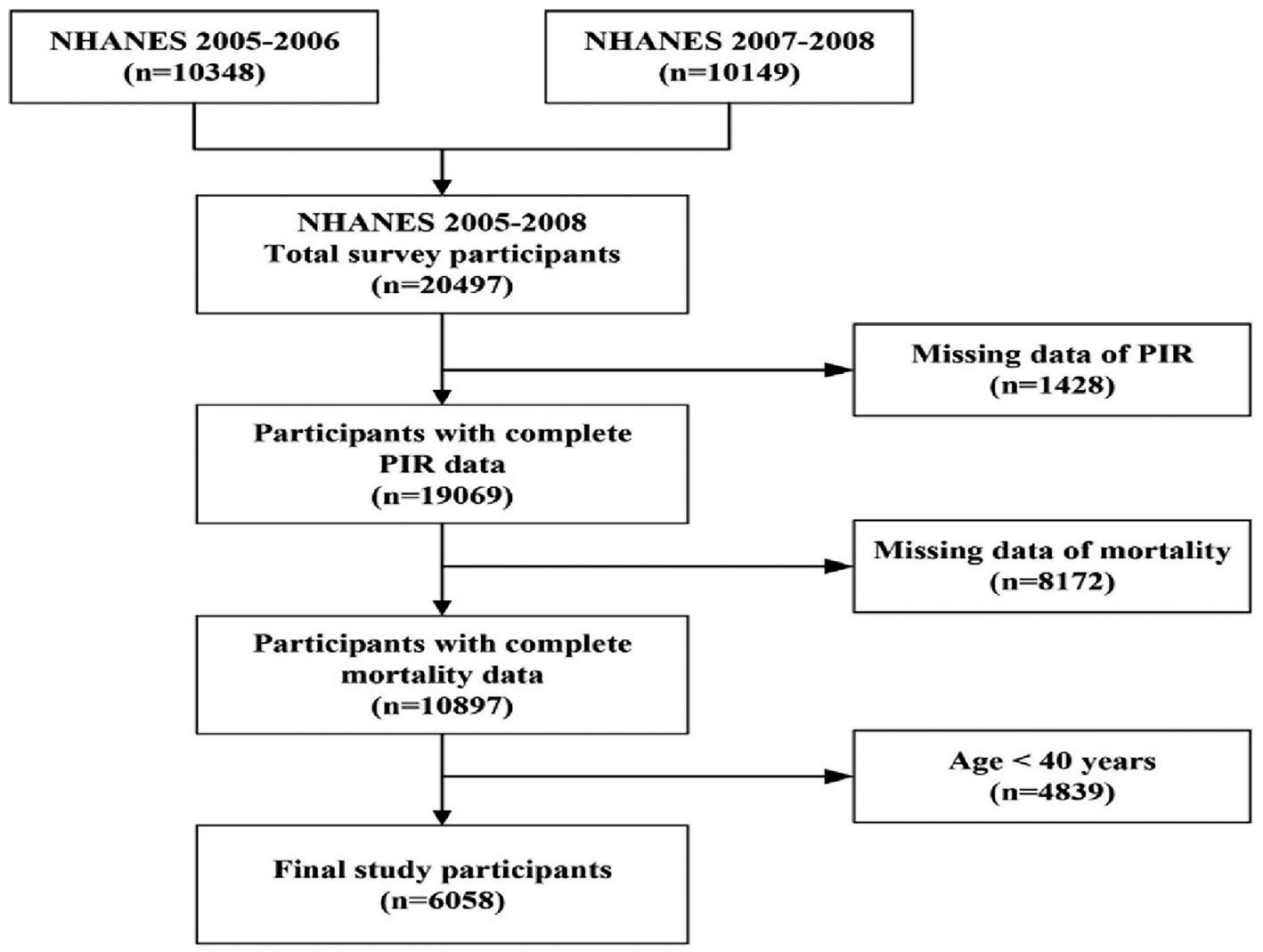
Flow chart of participants selection. NHANES, National Health and Nutrition Examination Survey; PIR, the ratio of family income to poverty.

This study utilized a prospective cohort design and employed data obtained from the US National Health and Nutrition Examination Survey (NHANES). The NHANES is a survey undertaken by the National Centre for Health Statistics (NCHS) of the United States. Centre for Disease Control and Prevention (CDC) is a large-scale, multistage, ongoing, and nationally representative health survey of noninstitutionalized US civilians in 2-year cycles since 1999–2000. The research study collects data on demographics, socioeconomic status, nutrition, physiology, and laboratory tests through a combination of interviews and medical exams. All participants provided written informed consent. In the current study, we used publically available data (https://www.cdc.gov/nchs/nhanes/index.htm) from two cycles of NHANES from 2005 to 2008 (2005–2006 and 2007–2008). The participants who did not meet the inclusion criteria were excluded from the analysis. This study initially included 20497 people. However, 6058 people were enrolled in the final analysis after the adoption of exclusion criteria. Figure 1 presents the study flow chart.

### Assessment of PIR

PIR is a predetermined continuous variable in NHANES. We utilized the PIR, which is an indicator of income relative to the economic needs of a household. This was achieved by calculating annual fluctuations in household size and cost of living and monitoring the consumer price index in relation to household income and federally established poverty limitations^16^. However, it is not applicable if respondents reported incomes below or above $20,000. Furthermore, values over 5.00 were coded as 5.00 or greater for confidentiality reasons. PIR levels were defined as low income (PIR<1), middle income (1≤PIR<4), and high income (PIR ≥ 4)^17^.

### Measurement of covariates

Covariates, such as age, sex, race/ethnicity, education, smoking status, alcohol intake, physical activity, sleep quality, self-reported diseases, health insurance and Body mass index (BMI) were required to account for spurious relationships between PRI and death. These data were obtained directly from the family questionnaire and medical conditions sections of the NHANES questionnaires. Non-Hispanic Whites, Blacks, Mexican Americans, and other racial groups were established^18^. The classification of marital status included married or cohabiting, widowed, divorced, separated, and never married^19^. There were three categories for educational level: less than high school, high school, and some college or higher^20^. We classified smokers in this study as either current smokers, former smokers (defined as 100 cigarettes or more smoked but no longer smoked), or never smokers (defined as less than 100 cigarettes smoked but no longer smoked)^18,21^. Participants were categorized into four alcohol consumption groups: never-drinkers, ex-drinkers, low-to-moderate drinkers (women consuming ⩽ 7 drinks/week or men consuming ⩽ 14 drinks/week), and heavy drinkers (women consuming > 7 drinks/week or men consuming > 14 drinks/week) according to standard guidelines^22,23^. Physical activity was classified into three groups: inactive (< 1 h*/* week of moderate or vigorous activity), moderately active (neither inactive nor active), and vigorous (> 2.5 h*/* week or > 1 h*/* week)^24^. When a household survey question was asked, "Are you covered by health insurance or any other type of health care plan?" and the response was affirmative, it was assumed that the individual had health insurance or some other type of healthcare coverage. The Pittsburgh Sleep Quality Index (PSQI) was developed to assess the quality and patterns of sleeping in adolescents or adults^25,26^. During NHANES 20052008, eight self-reported questions were used to determine PSQI^27^. Examining the PSQI score in sleep latency, sleep disturbance, and daytime dysfunction, sleep quality could be categorized as “poor” or “good”. The cumulative scores of the three components latency, disturbances, and daytime dysfunction—were utilized to derive the PSQI total score (0–23). A sleep quality score of five or higher indicated inadequate sleep, while a score below five was favorable^27^. The official NHANES website (https://www.cdc.gov/nchs/nhanes/) provides the interpretation, measurement, and calculation details for eight self-reported measures used to evaluate PSQI. BMI was determined by dividing the weight in kilograms by the square of the height in meters kg*/*m^2^. The World Health Organization (WHO) expert consultation used a BMI of 30 kg*/*m^2^ as the threshold to identify obesity^28,29^. The self-reported diseases were categorized as follows: diabetes, hypertension, hypercholesterolemia, cardiovascular disease, and other diseases. Diabetes diagnosis criteria include self-reported diabetes, medication or insulin use, HbAl > 6.5, and fasting glucose ⩾ 7.0^30^. If these people self-reported using antihypertensive drugs, they were diagnosed with hypertension; if they had a total cholesterol level of 6.2 mmol/L or more and were taking medication for it, they were diagnosed with hypercholesterolemia^29^. A self-reported medical history of coronary heart disease, myocardial infarction, angina, or stroke was considered cardiovascular disease (CVD)^31,32^. Additional ailments encompass self-reported conditions such as lung disease, arthritis, gout, thyroid disease, digestive system disease, and cancer.

### Ascertainment of mortality

This study investigated both identified and unidentified causes of mortality in order to determine the total death rate. Data on mortality were collected using the NHANES III linked mortality file, which was made available by the NCHS and the mortality assessment period lasted from December 31, 2015, to the baseline interview, which was carried out between 1988 and 1994^33^. From the survey date through December 31, 2015, we used data from the NHANES public-use linked mortality file (https://www.cdc.gov/nchs/data/datalinkage/LMF2015_Methodology_Analytic_Considerations.pdf), which was linked to the National Death Index using a probabilistic matching algorithm by the NCHS^34,35^.

### Statistical analysis

The differentiation between PIR classes was analyzed using one-way ANOVA and the χ^2^ test. The Cox proportional hazard regression model was utilized to determine the hazard ratios (HRs) for all-cause mortality in relation to PIR, along with a 95% confidence interval (CI). Personal time was measured based on the NHANES interview until death or the end of the follow-up period, whichever came first. Covariates and confounding factors were selected for their correlations with outcomes or effect estimates greater than 10%^36,37^. To address missing data in the covariates (all of which were categorical variables), we introduced a category specifically for missing data. This approach aimed to minimize the drop in sample size caused by missing covariate data^38^. Three models based on Cox regression analysis were used to determine the correlation between PIR and the risk of all-cause mortality. Model 1 did not consider any potential covariates. Demographics were considered when adjusting Model 2. All covariates were considered while adjusting Model 3. The test of Cochran-Armitage trend was used to test for a linear trend across various PIR levels. We used the likelihood ratio test to check for nonlinearity. The threshold effect of the PIR on all-cause mortality was examined using a smoothing function in a linear regression model. Furthermore, the one-line linear regression model was compared with the two-piecewise linear model using loglikelihood ratios. The analyses used Empower software (X&Y Solutions, Inc.; www.empowerstats.com) and statistical software R language (version 4.0.3), with statistically significant results when *P* < 0.05.

## Results

### Participant characteristics

The present analysis comprised 6058 participants (3057 men and 3001 females) aged 40–85 years from NHANES 2005 to 2008 (Fig. 1). In the low-income, middle-income, and high-income groups, there were 931, 3337, and 1790 participants, respectively. Table 1 shows the individuals in each of the three categories whose various demographic data differed considerably. The percentages of male participants (957 and 53.5%), non-Hispanic (Black or White) participants (1491 and 83.3%), and participants aged less than 65 years (1406 and 78.5%), married or living with a partner non-Hispanic (1378 and 77.0%), graduated from some college or more (1308 and 73.1%), smoking never or ever (935 and 52.2%; 608 and 34.0%), with low to moderate alcohol consumption (1135 and 69.5%), vigorous physical activity (667 and 43.6%), health insurance (1711 and 95.6%), hypercholesterolemia (2704 and 83.1%) were markedly higher in high-income level than other income levels. In comparison to other income levels, there were considerably more participants with diabetes (252 and 28.8%), cardiovascular disease (144 and 15.5%), and self-reported other conditions (739 and 79.4%) in low-income levels. In addition, a significantly higher proportion of individuals in the medium-income group (1583 and 47.5%) had hypertension than those in the lower-income group. In contrast, the average BMIs and percentages of good sleep were comparable across all three income levels.

**Table 1:** Evaluating indicators of different algorithms on regression tasks.

Characteristics	Total	Low income	Middle income	High income	*P*-value
	N = 6058	(PIR < 1*,* N = 931)	(PIR 1–4, N = 3337)	(*PIR* ⩾ 4*, N* = 1790)	
Age (years)					
< 65	4023 (66.4%)	625 (67.1%)	1992 (59.7%)	1406 (78.5%)	< 0.001
⩾ 65	2035 (33.6%)	306 (32.9%)	1345 (40.3%)	384 (21.5%)	
Gender, n (%)					
Male	3057 (50.5%)	435 (46.7%)	1665 (49.9%)	957 (53.5%)	0.002
Female	3001 (49.5%)	496 (53.3%)	1672 (50.1%)	833 (46.5%)	
Race/Ethnicity,n (%)					
Mexican American	970 (16.0%)	255 (27.4%)	562 (16.8%)	153 (8.5%)	< 0.001
Non-hispanic (Black, White)	4442 (73.3%)	540 (58.0%)	2411 (72.3%)	1491 (83.3%)	
Others	646 (10.7%)	136 (14.6%)	364 (10.9%)	146 (8.2%)	
Marital Status,n (%)					
Married or living with a partner	3860 (63.8%)	424 (45.7%)	2058 (61.7%)	1378 (77.0%)	< 0.001
Widowed, Divorced, or	1744 (28.8%)	381 (41.1%)	1042 (31.2%)	321 (17.9%)	
Separated					
Never married	449 (7.4%)	123 (13.3%)	236 (7.1%)	90 (5.0%)	
Education level, n (%)					
< High school	1850 (30.6%)	557 (60.0%)	1145 (34.3%)	148 (8.3%)	< 0.001
High school	1450 (24.0%)	172 (18.5%)	944 (28.3%)	334 (18.7%)	
Some college or more	2754 (45.5%)	200 (21.5%)	1246 (37.4%)	1308 (73.1%)	
Somking status, n (%)					< 0.001
Never	2866 (47.3%)	378 (40.8%)	1553 (46.6%)	935 (52.2%)	
Ever	1890 (31.2%)	222 (23.9%)	1060 (31.8%)	608 (34.0%)	
Current	1297 (21.4%)	327 (35.3%)	723 (21.7%)	247 (13.8%)	
Alcohol consumption,n (%)					< 0.001
Never drinkers	1111 (20.4%)	200 (24.4%)	660 (22.0%)	251 (15.4%)	
Ex-drinkers	1415 (25.9%)	289 (35.2%)	878 (29.3%)	248 (15.2%)	
Low-to-moderate drinkers	2916 (53.5%)	327 (39.8%)	1454 (48.5%)	1135 (69.5%)	
Heavy drinkers	12 (0.2%)	5 (0.6%)	7 (0.2%)	0 (0.0%)	
Physical activity, n (%)					< 0.001
Inactive	1059 (22.9%)	189 (31.4%)	643 (25.7%)	227 (14.8%)	
Moderate	1966 (42.5%)	250 (41.6%)	1081 (43.3%)	635 (41.5%)	
Vigorous	1604 (34.7%)	162 (27.0%)	775 (31.0%)	667 (43.6%)	
BMI (kg/m^2^)	29.4 ± 6.6	29.4 ± 7.7	29.5 ± 6.6	29.2 ± 6.0	0.528
Sleep quality, n (%)					0.168
Good sleep quality	2123 (35.4%)	302 (32.9%)	1197 (36.2%)	624 (35.1%)	
Poor sleep quality	3880 (64.6%)	617 (67.1%)	2110 (63.8%)	1153 (64.9%)	
Health insurance, n (%)					< 0.001
Yes	5041 (83.3%)	613 (66.0%)	2717 (81.5%)	1711 (95.6%)	
No	1011 (16.7%)	316 (34.0%)	616 (18.5%)	79 (4.4%)	
Diabetes, n (%)					< 0.001
Yes	1209 (21.2%)	252 (28.8%)	727 (23.3%)	230 (13.5%)	
No	4488 (78.8%)	624 (71.2%)	2395 (76.7%)	1469 (86.5%)	
Hypertension					< 0.001
Yes	2731 (45.1%)	429 (46.2%)	1583 (47.5%)	719 (40.2%)	
No	3318 (54.9%)	499 (53.8%)	1748 (52.5%)	1071 (59.8%)	
Hypercholesterolemia					< 0.001
Yes	4967 (84.0%)	626 (69.7%)	2704 (83.1%)	1637 (92.9%)	
No	948 (16.0%)	272 (30.3%)	550 (16.9%)	126 (7.1%)	
Cardiovascular disease					< 0.001
Yes	726 (12.0%)	144 (15.5%)	462 (13.9%)	120 (6.7%)	
No	5330 (88.0%)	787 (84.5%)	2873 (86.1%)	1670 (93.3%)	
self-reported other diseases					0.001
Yes	4650 (76.8%)	739 (79.4%)	2590 (77.6%)	1321 (73.8%)	
No	1408 (23.2%)	192 (20.6%)	747 (22.4%)	469 (26.2%)	

Data were expressed as mean ± standard deviation (SD) for for normally distributed continuous variables, and one-way ANOVAs were applied for multiple comparison. Categorical variables are reported in frequency and percentage and are compared with the use of the chi-square test. Slef-reported other diseases included respiratory disease, arthritis, gout, thyroid disease, digestive system disease, and cancer.

### Association between PIR and all-cause mortality

A total of 1445 patients experienced mortality from any cause, resulting in an incidence rate of 23.9%. The Cox regression analyses of all-cause mortality about PIR are presented in Table 2. We employed three models to assess the correlation between PIR and overall mortality. No covariates were considered in Model 1, whereas when revising Model 2, age, gender, and race/ethnicity were considered. Model 3 was adjusted all covariates presented in Table 1. In Model 3, after adjusting for various factors, the hazard ratios (HRs) for all-cause mortality were calculated for different income groups. Compared to the low-income group, the middle-income group had an HR of 0.80(95%CI 0.70–0.92), while the high-income group had an HR of 0.38 (95% CI 0.31–0.47). Model 3 shows that the middle-income and high-income groups had higher multivariateadjusted HRs (95% CIs) for all-cause mortality relative to the low-income group: 0.80 (95% CI 0.70 0.92) and 0.38(95% CI 0.31 0.47), respectively. when PIR considered as a categorical variable, and the results were similar. The trend test showed that, as the PIR value increases, the P value for PIR and all-cause mortality becomes increasingly more significant. These findings suggest that the PIR and all-cause mortality may not be linear when all factors are included.

**Table 2:** Crude and adjusted association between PIR and all-cause mortality in different models.

Exposure	Model 1		Model 2		Model 3	
	HR (95% CI)	*P* value	HR (95% CI)	*P* value	HR (95% CI)	*P* value
PIR classification	0.77 (0.74, 0.79)	< 0.001	0.76 (0.73, 0.79)	< 0.001	0.79 (0.76, 0.82)	< 0.001
Low income (PIR < 1)	1.00 (Reference)		1.00 (Reference)		1.00 (Reference)	
Middle income (PIR 1–4)	0.93 (0.81, 1.06)	0.264	0.72 (0.63, 0.82)	< 0.001	0.80 (0.70, 0.92)	0.002
High income (PIR⩾4) *P* for trend	0.33 (0.27, 0.39)	< 0.001	0.31 (0.25, 0.37)	< 0.001	0.38 (0.31, 0.47)	< 0.001
		< 0.001		< 0.001		< 0.001

Model 1: no covariates were adjusted. Model 2: age, gender, and race/ethnicity were adjusted. Model 3: all covariates presented in Table 1 were adjusted. A P value (P for trend) < 0.05 suggests that the linear trend is statistically significant. PIR, Ratio of family income to poverty.

### Nonlinearity and the threshold effect between PIR and all-cause mortality

**Figure 2:**
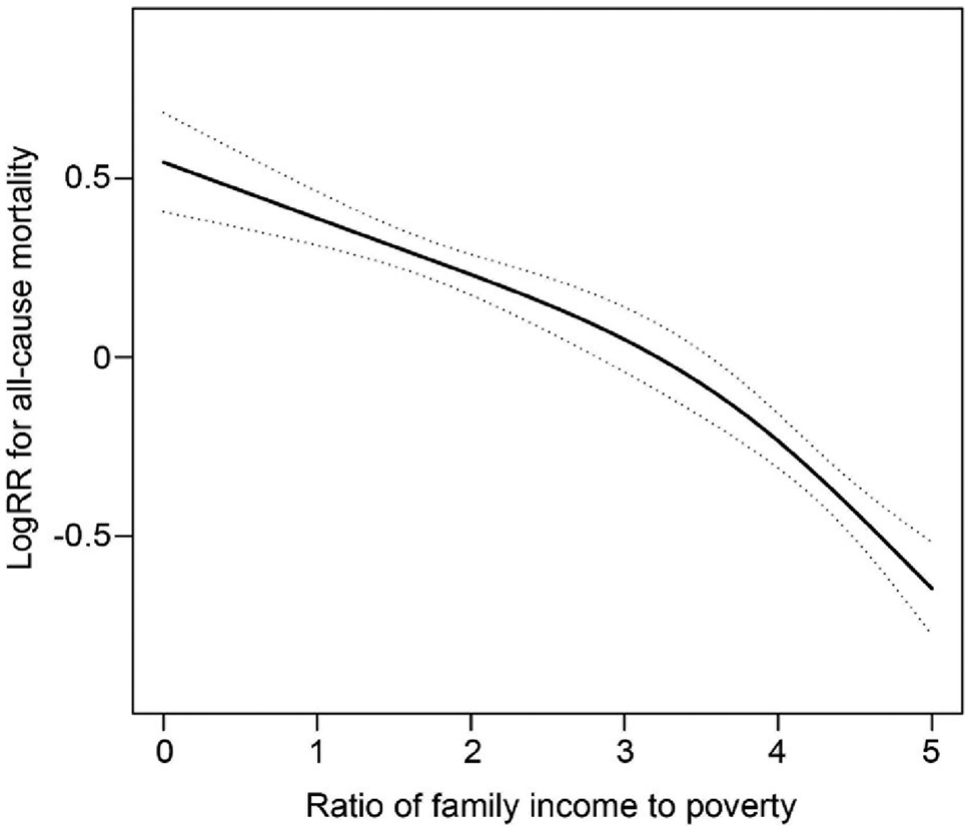
The smooth curve fitting showed that the non-linear association between PIR and all-cause mortality. All covariates were adjusted.

Even after accounting for all significant factors in Table 2, smooth curve fitting revealed a non-linear connection between PIR and all-cause mortality (Fig. 2). Two Cox proportional hazards regression models were used to fit the PIR-all-cause mortality relationship. The log-likelihood ratio test returned a *P*-value of <0.010. This suggests that the piecewise Cox proportional hazards regression model provides a more accurate explanation for PIR and all-cause mortality. The inflection point was determined to be at 3.5 using a combination of recursive and two-piecewise Cox proportional hazards regression. At PIR < 3.5, HR and 95% CI were 0.85 and 0.79*−*0.91, respectively. Table 3 shows that HR and 95% CI values for PIR ≥ 3.5 were 0.66 and 0.57*−*0.76, respectively.

**Table 3:** Crude and adjusted association between PIR and all-cause mortality in different models.

PIR	Model 1		Model 2		Model 3	
	HR (95% CI)	*P* value	HR (95% CI)	*P* value	HR (95% CI)	*P* value
PIR Inflection point	0.77 (0.74, 0.79)	< 0.001	0.76 (0.73, 0.79)	< 0.001	0.79 (0.76, 0.82)	< 0.001
< 3.5	0.88 (0.83, 0.94)	< 0.001	0.80 (0.75, 0.85)	< 0.001	0.85 (0.79, 0.91)	< 0.001
≥ 3.5	0.53 (0.46, 0.61)	< 0.001	0.66 (0.57, 0.75)	< 0.001	0.66 (0.57, 0.76)	< 0.001
P for log likelihood ratio test		< 0.001		0.034		0.010

Model 1: no covariates were adjusted. Model 2: age, gender, and race/ethnicity were adjusted. Model 3: all covariates presented in Table 1 were adjusted. PIR, Ratio of family income to poverty.

## Discussion

This study demonstrates the initial discovery of a non-linear correlation between PIR and mortality from any cause. This study indicates that the piecewise Cox proportional hazards regression model provides a more effective analysis of PIR and all-cause mortality. The P-value of the log-likelihood ratio test is < 0.010, which provides strong evidence to support this conclusion. We used a two-piecewise Cox proportional hazards regression and recursive technique to calculate the inflection point as 3.5. At PIR < 3.5, HR and 95% CI were 0.85 and 0.79 0.91, respectively. The HR and 95% CI for PIR 3.5 were 0.66 and 0.57 0.76, respectively.

Furthermore, this study found that individuals with a lower income had a higher likelihood of experiencing death from any cause, which aligns with the findings of subsequent analyses. Recent studies conducted in developed countries, such as the United States, have shown that there are lower odds ratios (ORs) for cardiovascular disease (CVD) risk among individuals with low and moderate incomes compared to those with high incomes. In contrast, lower SES was associated with greater mortality risk across all causes^39^. Zhang and colleagues investigated 1988–1994 (NHANES III) and 1999–2014 (continuous cycles) NHANES participants^39^. After determining SES by latent class analysis of PIR, employment, education, and health insurance, these researchers discovered that those with low SES were twice as likely to pass away from CVD-related causes and all causes combined. Gender differences have been observed in CHD death rates by Odutayo et al. Both genders had about double the CHD incidence in the low-income group as compared to the high-SES group^14^.

Possible mechanisms encompass a diverse array of resources, including knowledge, wealth, power, status, and supportive social networks. Additionally, protective factors such as access to health care services and a healthy lifestyle play a role. Other influential factors include education, medical compliance, stress levels, dietary habits, safety of neighbourhoods, physical activity, smoking and drug use, and air pollution^40–44^. For example, one study found that lifestyle variables explained 12.3% of the correlation between socioeconomic status and mortality^39^. People from poorer socioeconomic backgrounds are more likely to die from any cause due to a combination of biological, behavioural, and psychological risk factors^44^. The previously mentioned data clarifies the reason why individuals who have a low income are more prone to mortality from any cause.

There are various limitations in this study. Firstly, it is crucial to acknowledge that although the well-established PIR serves as the primary measure for income inequality in our research, it does not entirely consider the impact of SES on mortality from any cause. Secondly, it is critical to acknowledge the possibility of residual confounding because of the observational nature of this study. Lastly, we are unable to ascertain the direction or causation of the PIR-all-cause mortality connection due to the current cross-sectional study methodology.

## Conclusion

This study found a non-linear association between PIR and all-cause death among persons aged 40 and older. The PIR inflection point, where the link switches direction, was 3.5.

## Data Availability

Publicly available datasets were analyzed in this study. Tese data can be found at: www.cdc.gov/nchs/nhanes/.
